# Supplementary material for: Generalizability assessment of AI models across hospitals in a low-middle and high income country
Source: Nat Commun. 2024 Sep 27;15:8270. doi: 10.1038/s41467-024-52618-6 (PMC11436917; doi:10.1038/s41467-024-52618-6)
Supplement: Supplementary file 1 — Supplementary Information [file 41467_2024_52618_MOESM1_ESM.pdf]

Supplementary Material

Generalizability Assessment of AI Models Across Hospitals in a Low-Middle and High Income Country

Jenny Yang, Nguyen Thanh Dung, Pham Ngoc Thach, Nguyen Thanh Phong, Vu Dinh Phu, Khiem Dong Phu, Lam Minh Yen, Doan Bui Xuan Thy, Andrew A. S. Soltan, Louise Thwaites & David A. Clifton, 2024

A Software Packages and Implementation

Experiments were performed using Python (v3.8.3). All models were run using an Intel Xeon E-2146G Processor (CPU: 6 cores, 4.50 GHz max frequency). Logistic regression was implemented using Scikit-learn (v1.3.2). The XGBoost classifier was implemented using the XGBoost package (v2.0.3). Neural network models were implemented using Pytorch (2.1.2+cu121). Code can be found in: https://github.com/yangjenny/standard_algorithms

B Datasets

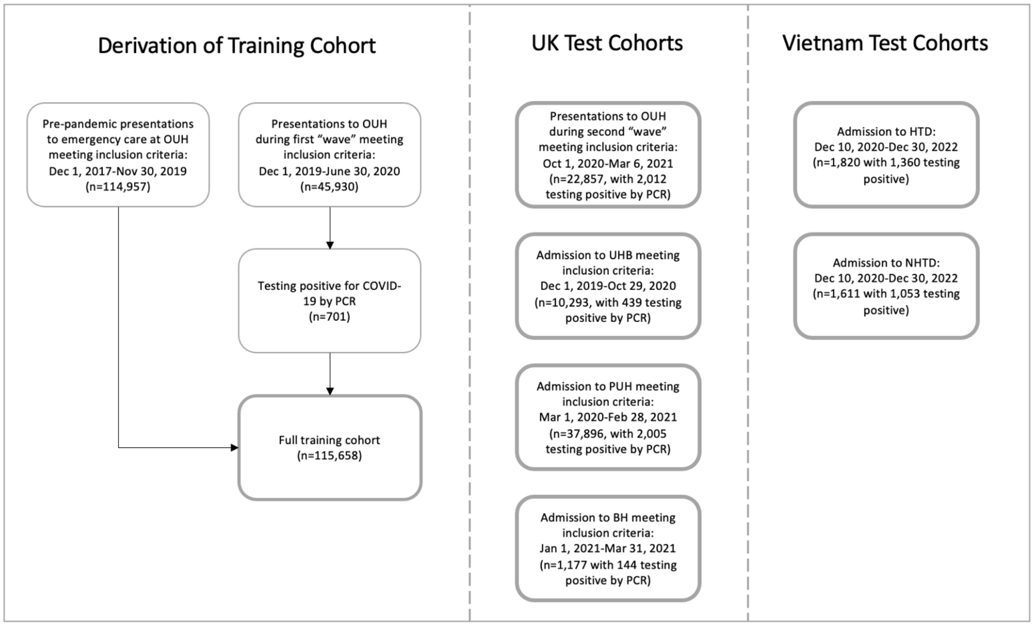

Supplementary Figure 1: Overview of datasets used during training and validation.

B.1 CURIAL Datasets

United Kingdom National Health Service (NHS) approval via the national oversight/regulatory body, the Health Research Authority (HRA), has been granted for development and validation of artificial intelligence models to detect Covid-19 (CURIAL; NHS HRA IRAS ID: 281832).

Data from OUH studied here are available from the Infections in Oxfordshire Research Database (https://oxfordbrc.nihr.ac.uk/research-themes/modernising-medical-microbiology-and-big-infection-diagnostics/infections-in-oxfordshire-research-database-iord/), subject to an application meeting the ethical and governance requirements of the Database. Data from UHB, PUH and BH are available on reasonable request to the respective trusts, subject to HRA requirements.

The following inclusions and exclusions are reproduced from previous studies [1,2,3].

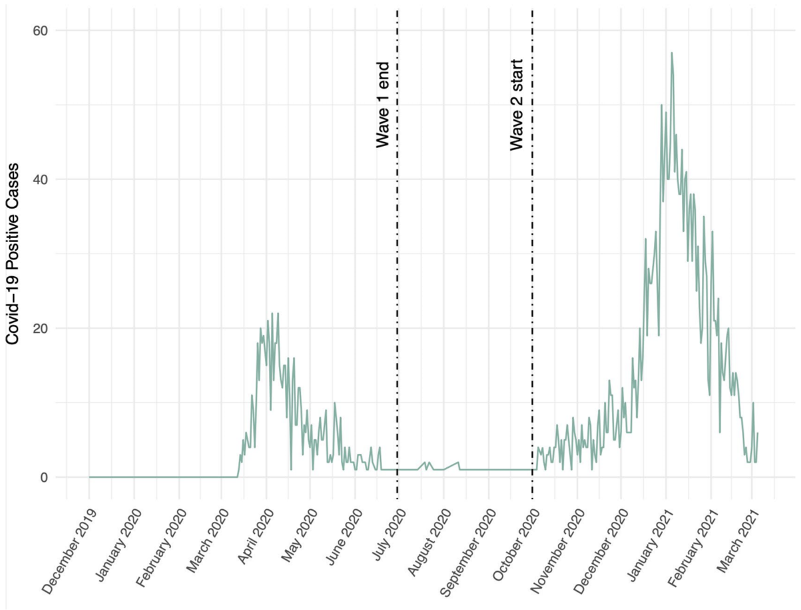

Supplementary Figure 2: Daily number of patients presenting to Oxford University Hospitals NHS Foundation Trust testing positive for COVID-19, between 1st December 2019 and 6th March 2021.

Oxford University Hospitals NHS Foundation Trust (OUH): We included all patients attending acute and emergency care settings at OUH who received routine blood tests on arrival, considering presentations before December 1, 2019, and thus before the pandemic, as the COVID-19-negative (control) cohort. We considered presentations during the 'first wave' of the UK COVID-19 pandemic (December 1, 2019 to June 30, 2020) with PCR confirmed SARS-CoV-2 infection as the COVID-19-positive (cases) cohort. We excluded patients who opted out of electronic health record (EHR) research and those who did not receive laboratory blood tests or were younger than 18 years of age. Due to incomplete penetrance of testing during the first wave of the pandemic, and imperfect sensitivity of the PCR test, there is uncertainty in the viral status of patients presenting during the pandemic who were untested or tested negative. We therefore selected a pre-pandemic control cohort during training to ensure absence of disease in patients labelled as COVID-19-negative. Clinical features extracted for each presentation included first-performed blood tests, blood gases, vital signs measurements and PCR testing for SARS-CoV-2 (Abbott Architect [Abbott, Maidenhead, UK], TaqPath [Thermo Fisher Scientific, Massachusetts, USA] and Public Health England-designed RNA-dependent RNA polymerase assays).

Portsmouth Hospitals University NHS Foundation Trust (PUH): PUH considered all patients admitted to the Queen Alexandria Hospital, serving a population of 675,000 and offering tertiary referral services to the surrounding region, between March 1, 2020 and February 28, 2021. Confirmatory COVID-19 testing was by laboratory SARS-CoV2 RT-PCR assay, considering any positive PCR result within 48hrs of admission as a true positive.

University Hospitals Birmingham NHS Foundation Trust (UHB): UHB considered all patients admitted to The Queen Elizabeth Hospital, Birmingham, between December 01, 2019 and October 29, 2020. The Queen Elizabeth Hospital is a large tertiary referral unit within the UHB group which provides healthcare services for a population of 2.2 million across the West Midlands. Confirmatory COVID-19 testing was performed by laboratory SARS-CoV-2 RT-PCR assay.

Bedfordshire NHS Foundation Trust (BH): BH considered all patients admitted to Bedford Hospital between January 1, 2021 and March 31, 2021. BH provides healthcare services for a population of around 620,000 in Bedfordshire. Confirmatory COVID-19 testing was performed on the day of admission by point-of-care PCR based nucleic acid testing [SAMBA-II & Panther Fusion System, Diagnostics in the Real World, UK, and Hologic, USA].

B.2 Vietnam Datasets

The ethics committees of the Hospital for Tropical Diseases (HTD) and the National Hospital for Tropical Diseases (NHTD) approved use of the HTD and NHTD datasets for COVID-19 diagnosis, respectively.

Hospital for Tropical Diseases (HTD): HTD considered all patients admitted between January 1, 2021 and December 31,2022. Confirmatory COVID-19 testing was performed using PCR.

National Hospital for Tropical Diseases (NHTD): NHTD considered all patients admitted between January 1, 2021 and December 31,2022. Confirmatory COVID-19 testing was performed using PCR and/or rapid antigen testing.

Supplementary Table 1: Diagnostic testing method for COVID-19 detection.

COVID-19 Test-type	NHTD
PCR	880
Rapid Antigen Test	69
Other	58
Unspecified	604

Supplementary Table 2: Distribution of vital signs, reported as median and interquartile ranges, for each patient cohort.

	Oxford University Hospitals (pre-pandemic & wave one cases, to June 30/20)		Oxford University Hospitals	Portsmouth Hospitals University NHS Trust	University Hospitals Birmingham NHS Foundation Trust	Bedfordshire Hospitals NHS Foundation Trust	Hospital for Tropical Diseases	National Hospital for Tropical Diseases	Kruskal-Wallis, p-value
	Prepandemic	COVID-19-cases	Oct 1/20-Mar 6/21	Mar 1/20-Feb 28/21	Dec 1/19 - Oct 29/20	Jan 1/21-Mar 31/21	Jan 1/21-Dec 31/22	Jan 1/21-Dec 31/22	
Respiratory Rate (breath/min)	18.0 (16.0-19.0)	20.0 (18.0-24.0)	18.0 (16.6-19.0)	17.0 (16.0-19.0)	18.0 (17.0-20.0)	18.0 (16.0-20.0)	24.0(20.0-26.5)	25.0(22.0-30.0)	<0.0001
Heart Rate (beats/min)	82.0 (71.0-96.0)	88.0 (75.0-101.0)	84.0 (72.0-97.0)	82.0 (71.0-95.0)	86.0 (73.0-101.0)	84.0 (73.0-97.0)	94.0(84.0-108.0)	99.0(86.0-114.0)	<0.0001
Systolic Blood Pressure (mmHg)	132.0 (118.0-150.0)	131.0 (115.0-146.0)	134.0 (119.0-152.0)	128.0 (114.0-146.0)	136.0 (119.0-155.0)	131.0 (116.0-149.0)	130.0(111.5-140.0)	120.0(110.0-136.0)	<0.0001
Diastolic Blood Pressure (mmHg)	74.0 (65.0-84.0)	74.0 (64.0-84.0)	75.0 (65.0-85.0)	76.0 (67.0-84.0)	77.0 (68.0-87.0)	78.0 (68.0-88.0)	80.0(70.0-80.0)	70.0(60.0-80.0)	<0.0001
Tympanic Temperature (C)	36.5 (36.1-36.9)	36.9 (36.3-37.6)	36.3 (36.0-36.7)	36.3 (36.0-36.8)	36.7 (36.4-37.2)	36.5 (36.4-36.9)	37.0(37.0-37.3)	37.0(36.8-37.5)	<0.0001

Supplementary Table 3: Distribution of blood test features, reported as median and interquartile ranges, for each patient cohort.

	Oxford University Hospitals (pre-pandemic & wave one cases, to June 30/20)		Oxford University Hospitals	Portsmouth Hospitals University NHS Trust	University Hospitals Birmingham NHS Foundation Trust	Bedfordshire Hospitals NHS Foundation Trust	Hospital for Tropical Diseases	National Hospital for Tropical Diseases	Kruskal-Wallis, p-value
	Prepandemic cohort	COVID-19-cases cohort	Oct 1/20-Mar 6/21	Mar 1/20-Feb 28/21	Dec 1/19 - Oct 29/20	Jan 1/21-Mar 31/21	Jan 1/21-Dec 31/22	Jan 1/21-Dec 31/22	
HAEMOGLOBIN (g/L)	130.0 (116.0-142.0)	130.0 (114.0-144.0)	129.0 (114.0-142.0)	129.0 (114.0-143.0)	127.0 (113.0-140.0)	134.0 (119.0-146.0)	128.0(113.0-141.0)	112.0(95.0-127.0)	<0.0001
WHITE CELLS (10^91^{-1})	8.45 (6.46-11.18)	6.98 (5.14-9.72)	8.94 (6.7-12.06)	8.6 (6.7-11.3)	9.4 (7.1-12.6)	9.2 (6.9-12.5)	9.55(6.69-13.565)	10.9(7.525-15.0)	<0.0001
PLATELETS (10^91^{-1})	249.0 (199.0-307.0)	215.0 (163.0-283.5)	251.0 (198.0-314.0)	251.0 (199.0-312.0)	247.0 (196.0-311.0)	246.0 (196.0-310.0)	216.0(152.0-281.0)	189.5(116.75-260.25)	<0.0001
HAEMATOCRIT	0.39 (0.35-0.42)	0.4 (0.35-0.44)	0.39 (0.35-0.43)	0.39 (0.34-0.42)	0.38 (0.34-0.42)	0.39 (0.35-0.43)	0.392(0.35-0.428)	0.342(0.289-0.387)	<0.0001
SODIUM (mM)	138.0 (136.0-140.0)	136.0 (134.0-139.0)	138.0 (135.0-140.0)	138.0 (136.0-140.0)	137.0 (134.0-139.0)	138.0 (136.0-140.0)	134.0(130.0-137.0)	136.0(132.65-139.65)	<0.0001
UREA (mM)	5.3 (4.0-7.4)	5.9 (4.2-9.07)	5.7 (4.2-8.3)	5.2 (3.8-7.6)	6.2 (4.5-9.0)	5.8 (4.2-8.3)	7.5(5.0-12.75)	7.215(4.8-11.7)	<0.0001
BILIRUBIN (umol/L)	9.0 (6.0-13.0)	9.0 (7.0-13.25)	9.0 (6.0-14.0)	10.0 (7.0-16.0)	10.0 (7.0-15.0)	10.0 (7.0-14.0)	13.7(7.8-36.7)	9.8(6.4-16.375)	<0.0001
CREATININE (umol/L)	73.0 (60.0-93.0)	79.0 (65.0-106.0)	74.0 (60.0-97.0)	74.0 (60.0-96.0)	78.0 (62.0-105.0)	80.5 (65.75-104.0)	76.0(61.0-99.0)	77.65(56.0-121.0)	<0.0001
POTASSIUM (mM)	4.0 (3.7-4.3)	4.0 (3.7-4.3)	4.0 (3.8-4.4)	4.2 (3.9-4.4)	4.1 (3.8-4.4)	4.3 (4.0-4.6)	3.72(3.35-4.09)	3.8(3.4-4.3)	<0.0001
MEAN CELL VOL (fl)	89.6 (86.0-93.4)	90.2 (86.6-94.2)	90.2 (86.6-94.2)	89.0 (84.9-93.0)	89.9 (86.2-93.6)	88.0 (85.0-92.0)			0.210
NEUTROPHILS (10^91^{-1})	5.72 (3.99-8.36)	5.11 (3.48-7.49)	6.44 (4.4-9.55)	5.9 (4.2-8.6)	6.9 (4.7-10.0)	6.8 (4.7-9.73)			<0.0001
LYMPHOCYTES (10^91^{-1})	1.51 (1.0-2.13)	0.96 (0.65-1.38)	1.31 (0.85-1.89)	1.5 (0.97-2.2)	1.3 (0.9-1.9)	1.27 (0.86-1.83)			<0.0001
MONOCYTES (10^91^{-1})	0.64 (0.48-0.85)	0.49 (0.35-0.74)	0.66 (0.48-0.89)	0.63 (0.48-0.85)	0.7 (0.5-0.9)	0.66 (0.48-0.92)			<0.0001
EOSINOPHILS (10^91^{-1})	0.1 (0.04-0.2)	0.01 (0.0-0.06)	0.07 (0.02-0.16)	0.1 (0.02-0.2)	0.1 (0.0-0.2)	0.06 (0.02-0.16)			<0.0001
BASOPHILS (10^91^{-1})	0.04 (0.03-0.06)	0.02 (0.01-0.03)	0.04 (0.02-0.06)	0.04 (0.02-0.06)	0.1 (0.0-0.1)	0.05 (0.03-0.07)			<0.0001
ALBUMIN (g/L)	36.0 (32.0-39.0)	32.0 (28.0-35.0)	36.0 (31.0-39.0)	36.0 (31.0-40.0)	36.0 (32.0-40.0)	35.0 (31.0-39.0)			0.006
ALKALINE PHOSPHATASE (IU/L)	80.0 (64.0-105.0)	82.0 (64.0-108.0)	84.0 (66.0-112.0)	84.0 (67.0-109.0)	90.0 (71.0-119.0)	94.0 (74.5-122.0)			<0.0001
ALT (IU/L)	18.0 (13.0-28.0)	25.0 (17.0-41.0)	20.0 (13.0-33.0)	19.0 (13.0-30.0)	19.0 (13.0-30.0)	20.0 (13.0-31.0)			<0.0001
eGFR (ml/min)	85.0 (63.0-150.0)	78.0 (53.0-150.0)	84.0 (58.0-150.0)	83.0 (60.0-90.0)	76.0 (52.0-90.0)	76.0 (54.0-90.0)			0.011
CRP (mg/L)	8.6 (2.3-39.0)	72.5 (23.8-143.6)	15.8 (3.5-67.4)	13.0 (3.0-71.0)	12.0 (3.0-61.0)	10.7 (2.8-48.78)			0.003

C Model Architectures

Neural Network Models: The rectified linear unit (ReLU) activation function was used for the hidden layers and the Sigmoid activation function was used in the output layer. For updating model weights, binary cross entropy loss and the Adaptive Moment Estimation (Adam) optimizer was used during training. Models were trained using 100 epochs and a batch size of 2048. Add summary of model

```
CovidClassifier(
  (hidden1): Linear(in_features=14, out_features=10, bias=True)
  (act1): ReLU()
  (output): Linear(in_features=10, out_features=1, bias=True)
  (act_output): Sigmoid()
)
```

Supplementary Figure 3: Neural network architecture used in COVID-19 classification task.

D Results

Supplementary Table 4: COVID-19 diagnosis performance across logistic regression, XGBoost, and neural network models trained on the UK data. Results are presented for the reduced feature set. Results are optimized to sensitivities of 0.85. Bolded values denote the best scores across models for each test set. Metrics are reported alongside 95% confidence intervals (CIs). Source data are provided as a Source Data file.

Test Set	Model	AUROC	AUPRC	Sensitivity	Specificity	PPV	NPV
OUH	LR	0.795(0.785-0.805)	0.35(0.334-0.37)	0.692(0.676-0.709)	**0.79(0.785-0.794)**	**0.241(0.235-0.247)**	**0.964(0.962-0.966)**
	XGB	0.784(0.774-0.793)	0.408(0.388-0.426)	0.711(0.693-0.727)	0.716(0.711-0.721)	0.195(0.19-0.199)	0.962(0.96-0.964)
	NN	**0.803(0.793-0.812)**	**0.428(0.408-0.448)**	**0.718(0.701-0.733)**	0.725(0.72-0.73)	0.201(0.197-0.206)	**0.964(0.962-0.966)**
PUH	LR	0.812(0.803-0.821)	0.222(0.209-0.235)	0.781(0.765-0.796)	0.696(0.692-0.7)	0.126(0.123-0.128)	0.983(0.981-0.984)
	XGB	**0.817(0.808-0.825)**	**0.346(0.326-0.365)**	0.766(0.75-0.781)	**0.709(0.704-0.712)**	**0.128(0.125-0.131)**	0.982(0.981-0.983)
	NN	0.816(0.808-0.825)	0.307(0.29-0.326)	**0.835(0.822-0.849)**	0.592(0.587-0.596)	0.103(0.101-0.104)	**0.985(0.983-0.986)**
UHB	LR	0.757(0.735-0.782)	0.196(0.169-0.228)	0.599(0.562-0.64)	**0.821(0.815-0.827)**	**0.129(0.122-0.138)**	0.979(0.977-0.981)
	XGB	0.76(0.74-0.78)	0.191(0.163-0.225)	0.617(0.58-0.656)	0.754(0.747-0.762)	0.101(0.095-0.107)	0.978(0.976-0.98)
	NN	**0.776(0.755-0.797)**	**0.242(0.21-0.279)**	**0.69(0.654-0.726)**	0.743(0.736-0.75)	0.107(0.102-0.113)	**0.982(0.98-0.984)**
BH	LR	0.801(0.768-0.833)	0.363(0.308-0.433)	0.549(0.48-0.616)	**0.861(0.842-0.878)**	**0.354(0.314-0.395)**	0.932(0.922-0.941)
	XGB	0.773(0.735-0.807)	0.409(0.344-0.481)	0.576(0.508-0.646)	0.811(0.791-0.831)	0.299(0.265-0.332)	0.932(0.922-0.943)
	NN	**0.804(0.772-0.838)**	**0.442(0.377-0.519)**	**0.688(0.619-0.754)**	0.74(0.717-0.763)	0.269(0.244-0.296)	**0.944(0.933-0.956)**
HTD	LR	0.488(0.465-0.515)	0.717(0.694-0.74)	0.896(0.883-0.91)	0.1(0.077-0.124)	0.746(0.741-0.752)	0.246(0.196-0.295)
	XGB	0.533(0.508-0.559)	0.755(0.732-0.779)	0.803(0.786-0.82)	**0.202(0.172-0.236)**	0.748(0.741-0.757)	0.258(0.227-0.293)
	NN	**0.577(0.551-0.604)**	**0.763(0.741-0.786)**	**0.908(0.895-0.921)**	0.139(0.114-0.167)	**0.757(0.751-0.764)**	**0.339(0.287-0.395)**
NHTD	LR	0.471(0.446-0.497)	0.631(0.606-0.66)	0.824(0.805-0.843)	0.161(0.136-0.187)	0.65(0.641-0.659)	0.327(0.284-0.372)
	XGB	0.478(0.453-0.504)	0.633(0.608-0.663)	0.727(0.703-0.75)	**0.272(0.242-0.304)**	**0.654(0.641-0.666)**	**0.346(0.313-0.379)**
	NN	**0.515(0.491-0.541)**	**0.671(0.647-0.7)**	**0.831(0.812-0.85)**	0.159(0.134-0.185)	0.651(0.642-0.66)	0.333(0.288-0.377)

Supplementary Table 5: COVID-19 diagnosis performance across logistic regression, XGBoost, and neural network models trained on the UK data. Results are presented for the comprehensive (GATS-filled) dataset. Results are optimized to sensitivities of 0.85. Bolded values denote the best scores across models for each test set. Metrics are reported alongside 95% confidence intervals (CIs). Source data are provided as a Source Data file.

Test Set	Model	AUROC	AUPRC	Sensitivity	Specificity	PPV	NPV
OUH	LR	0.854(0.845-0.862)	0.472(0.452-0.493)	0.738(0.721-0.754)	**0.85(0.846-0.854)**	**0.322(0.314-0.329)**	0.971(0.969-0.973)
	XGB	**0.877(0.869-0.885)**	**0.636(0.618-0.654)**	**0.815(0.799-0.829)**	0.774(0.769-0.778)	0.258(0.252-0.263)	**0.977(0.976-0.979)**
	NN	0.865(0.856-0.872)	0.587(0.568-0.606)	0.77(0.753-0.785)	0.8(0.795-0.804)	0.271(0.265-0.277)	0.973(0.971-0.975)
PUH	LR	0.832(0.823-0.84)	0.308(0.292-0.325)	0.759(0.744-0.774)	**0.753(0.75-0.757)**	0.147(0.144-0.15)	0.982(0.981-0.984)
	XGB	**0.877(0.869-0.884)**	**0.506(0.486-0.525)**	**0.825(0.81-0.839)**	0.748(0.744-0.751)	**0.154(0.151-0.158)**	**0.987(0.986-0.988)**
	NN	0.853(0.844-0.861)	0.433(0.414-0.454)	0.786(0.771-0.801)	**0.753(0.75-0.757)**	0.151(0.148-0.154)	0.984(0.983-0.985)
UHB	LR	0.846(0.828-0.864)	0.297(0.264-0.34)	0.747(0.712-0.782)	**0.832(0.826-0.839)**	**0.166(0.158-0.174)**	0.987(0.985-0.988)
	XGB	**0.86(0.845-0.876)**	0.38(0.342-0.422)	**0.779(0.745-0.812)**	0.787(0.781-0.794)	0.14(0.134-0.147)	**0.988(0.986-0.989)**
	NN	0.859(0.841-0.876)	**0.386(0.347-0.431)**	0.772(0.737-0.803)	0.795(0.788-0.802)	0.144(0.137-0.151)	0.987(0.985-0.989)
BH	LR	0.883(0.856-0.908)	0.519(0.454-0.597)	0.66(0.594-0.721)	**0.918(0.904-0.932)**	**0.528(0.482-0.58)**	0.951(0.942-0.959)
	XGB	**0.905(0.881-0.926)**	**0.669(0.607-0.729)**	**0.792(0.732-0.848)**	0.849(0.83-0.867)	0.422(0.39-0.46)	**0.967(0.958-0.976)**
	NN	0.875(0.846-0.902)	0.579(0.51-0.654)	0.688(0.623-0.747)	0.873(0.856-0.891)	0.43(0.391-0.472)	0.952(0.943-0.961)
HTD	LR	0.492(0.466-0.519)	0.728(0.706-0.752)	0.571(0.549-0.593)	0.435(0.396-0.474)	0.749(0.735-0.764)	0.255(0.236-0.276)
	XGB	0.539(0.514-0.566)	0.774(0.752-0.798)	0.646(0.625-0.668)	0.404(0.367-0.44)	0.762(0.749-0.774)	0.278(0.256-0.299)
	NN	**0.59(0.563-0.617)**	**0.784(0.762-0.808)**	**0.661(0.64-0.682)**	**0.465(0.426-0.505)**	**0.785(0.772-0.799)**	**0.317(0.295-0.34)**
NHTD	LR	0.475(0.449-0.498)	0.641(0.616-0.671)	0.54(0.516-0.564)	**0.432(0.397-0.465)**	0.642(0.624-0.658)	0.332(0.311-0.352)
	XGB	0.484(0.458-0.508)	0.642(0.617-0.671)	0.61(0.585-0.633)	0.369(0.335-0.404)	0.646(0.632-0.662)	0.334(0.31-0.359)
	NN	**0.522(0.497-0.544)**	**0.68(0.655-0.707)**	**0.66(0.635-0.682)**	0.353(0.319-0.385)	**0.658(0.644-0.672)**	**0.355(0.328-0.382)**

Supplementary Table 6: COVID-19 diagnosis performance across neural network models, trained using transfer learning at HTD and NHTD centres. Models are evaluated prospectively on the site used for transfer learning (remaining subset not used in training) and externally on the other site (full dataset). Results are presented for both the reduced feature set, and the comprehensive feature set. Sensitivity is optimized to 0.85. Metrics are reported alongside 95% confidence intervals (CIs). Source data are provided as a Source Data file.

Features	Training Set	Test Set	AUROC	AUPRC	Sensitivity	Specificity	PPV	NPV
Reduced	HTD	HTD	0.707(0.654-0.756)	0.769(0.715-0.828)	0.876(0.841-0.911)	0.418(0.342-0.491)	0.749(0.725-0.775)	0.63(0.557-0.706)
		NHTD	0.653(0.627-0.677)	0.748(0.723-0.776)	0.855(0.837-0.874)	0.328(0.294-0.361)	0.706(0.695-0.718)	0.545(0.505-0.586)
	NHTD	HTD	0.656(0.599-0.712)	0.818(0.77-0.869)	0.816(0.773-0.855)	0.386(0.3-0.471)	0.78(0.755-0.804)	0.442(0.365-0.52)
		NHTD	0.65(0.623-0.675)	0.815(0.795-0.84)	0.801(0.785-0.819)	0.422(0.385-0.46)	0.804(0.793-0.815)	0.418(0.388-0.448)
Full	HTD	HTD	0.727(0.676-0.774)	0.792(0.745-0.845)	0.897(0.863-0.926)	0.467(0.391-0.541)	0.77(0.744-0.795)	0.695(0.621-0.77)
		NHTD	0.663(0.639-0.686)	0.758(0.732-0.785)	0.877(0.861-0.895)	0.269(0.236-0.299)	0.694(0.684-0.703)	0.538(0.497-0.583)
	NHTD	HTD	0.668(0.61-0.722)	0.836(0.794-0.877)	0.769(0.721-0.813)	0.489(0.402-0.575)	0.8(0.772-0.829)	0.443(0.378-0.513)
		NHTD	0.69(0.666-0.713)	0.845(0.826-0.868)	0.747(0.728-0.766)	0.524(0.486-0.56)	0.823(0.811-0.835)	0.412(0.387-0.438)

Supplementary Table 7: COVID-19 diagnosis performance using locally-trained models. Results are presented for neural network models, trained using a subset of data from either the HTD or NHTD centres. Models are evaluated prospectively on the site used for model development (i.e. remaining subset not used in training) and externally on the other site (full dataset). Sensitivity is optimized to 0.85. Metrics are reported alongside 95% confidence intervals (CIs). Source data are provided as a Source Data file.

Training Set	Test Set	AUROC	AUPRC	Sensitivity	Specificity	PPV	NPV
HTD	HTD	0.664(0.613-0.716)	0.75(0.699-0.806)	0.868(0.833-0.904)	0.369(0.296-0.436)	0.732(0.709-0.755)	0.584(0.506-0.664)
	NHTD	0.639(0.615-0.663)	0.739(0.714-0.768)	0.849(0.831-0.868)	0.305(0.271-0.338)	0.697(0.687-0.709)	0.517(0.476-0.559)
NHTD	HTD	0.608(0.585-0.634)	0.808(0.788-0.832)	0.76(0.741-0.78)	0.378(0.342-0.415)	0.783(0.773-0.794)	0.348(0.322-0.377)
	NHTD	0.662(0.604-0.717)	0.829(0.784-0.873)	0.786(0.742-0.83)	0.455(0.366-0.542)	0.793(0.766-0.822)	0.444(0.374-0.515)

HTD – outcome distributions

Neural Network, reduced feature set

Class 0 – COVID-negative

Class 1 – COVID-positive

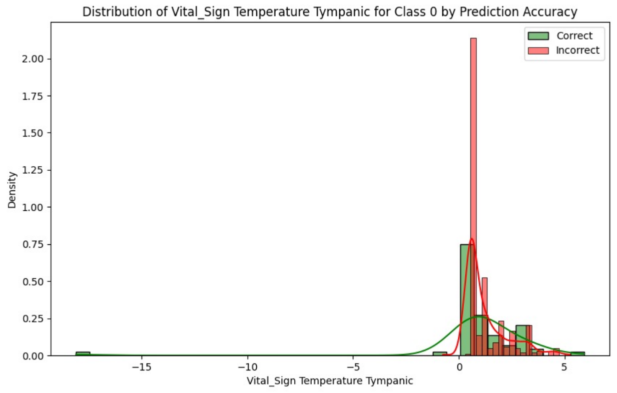

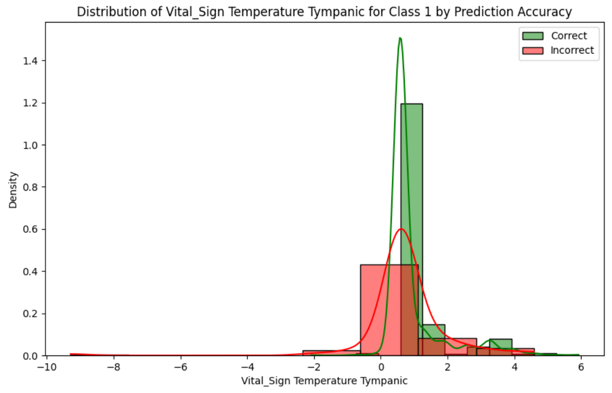

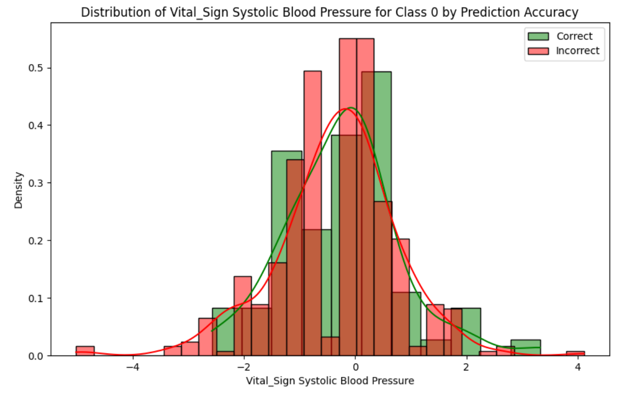

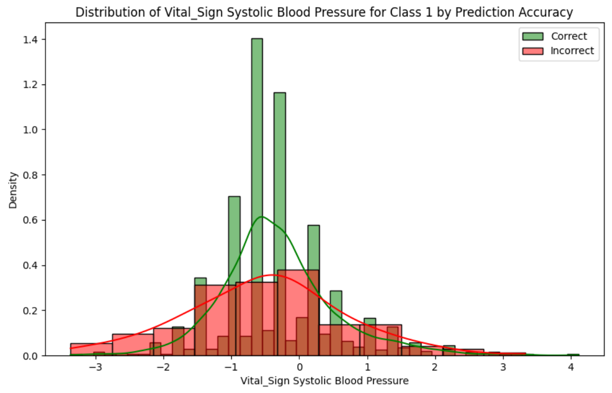

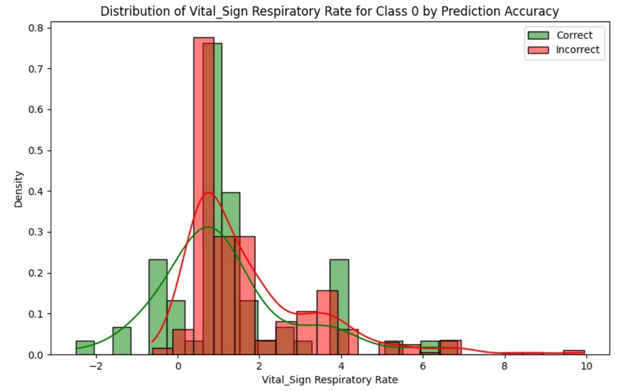

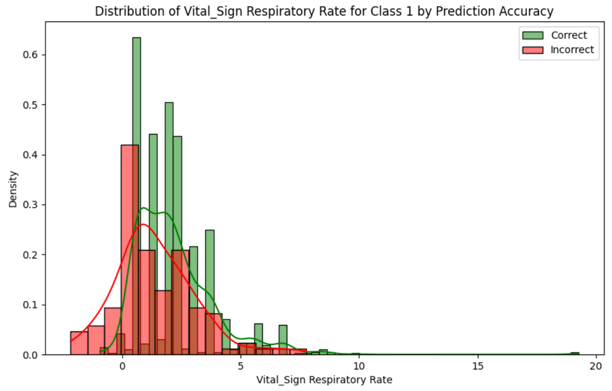

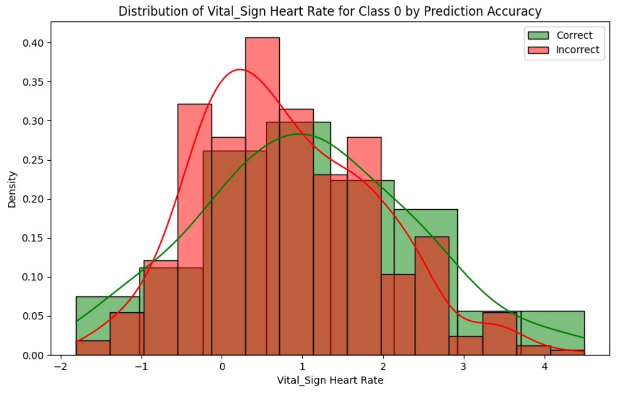

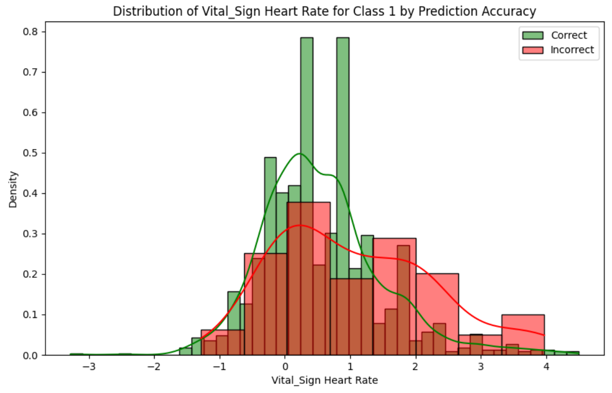

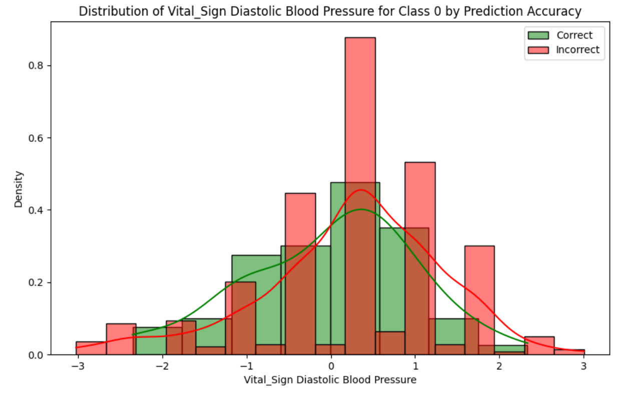

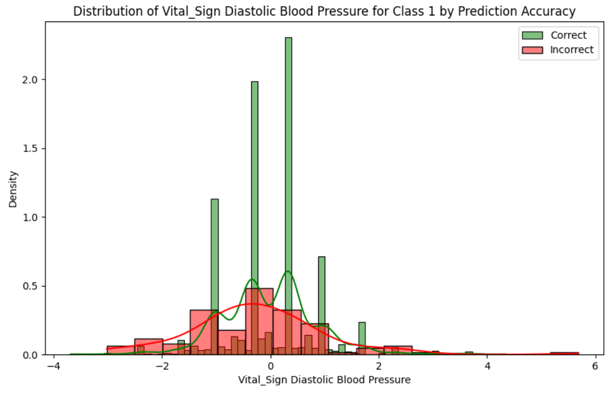

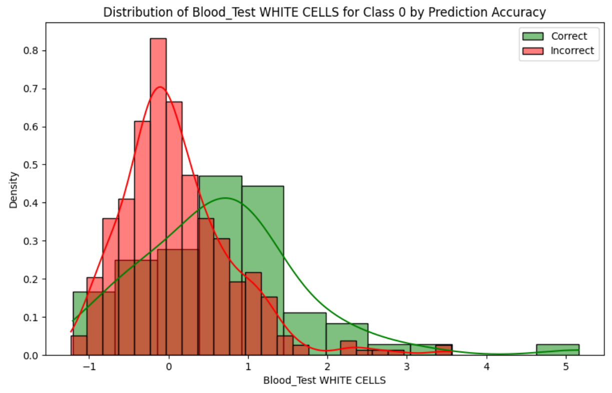
Distribution of Blood_Test WHITE CELLS for Class 0 by Prediction Accuracy
Correct
Incorrect
Density
Blood_Test WHITE CELLS

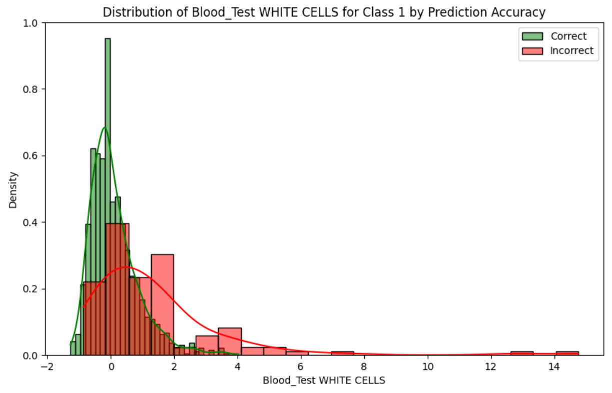
Distribution of Blood_Test WHITE CELLS for Class 1 by Prediction Accuracy
Correct
Incorrect
Density
Blood_Test WHITE CELLS

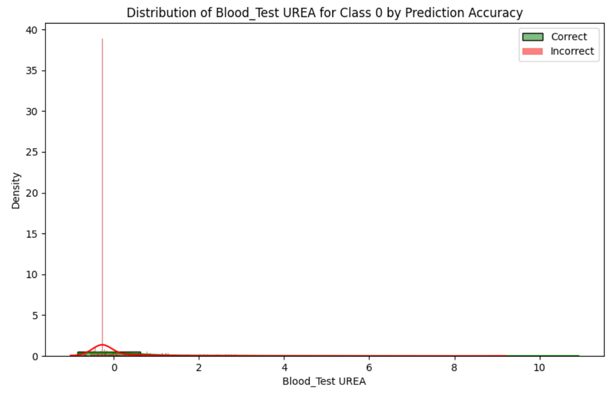
Distribution of Blood_Test UREA for Class 0 by Prediction Accuracy
Correct
Incorrect
Density
Blood_Test UREA

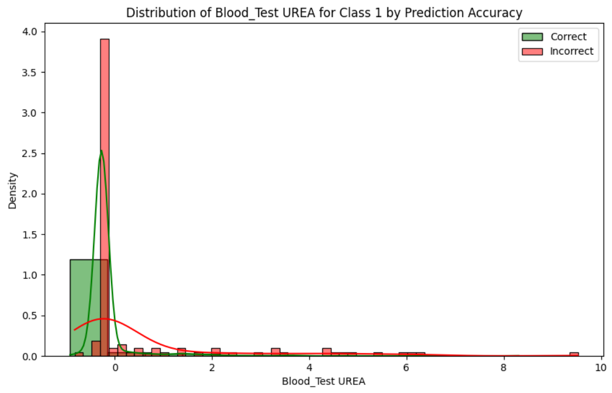
Distribution of Blood_Test UREA for Class 1 by Prediction Accuracy
Correct
Incorrect
Density
Blood_Test UREA

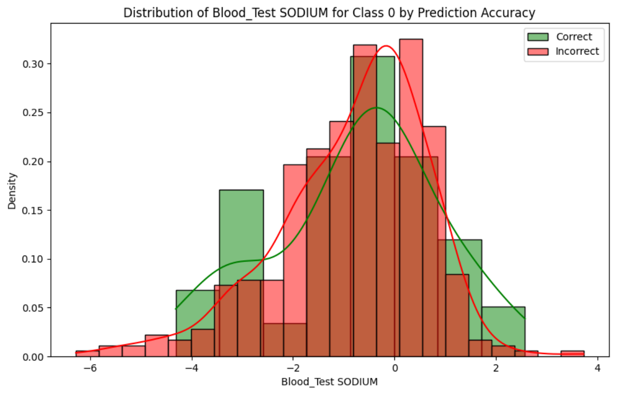
Distribution of Blood_Test SODIUM for Class 0 by Prediction Accuracy
Correct
Incorrect
Density
Blood_Test SODIUM

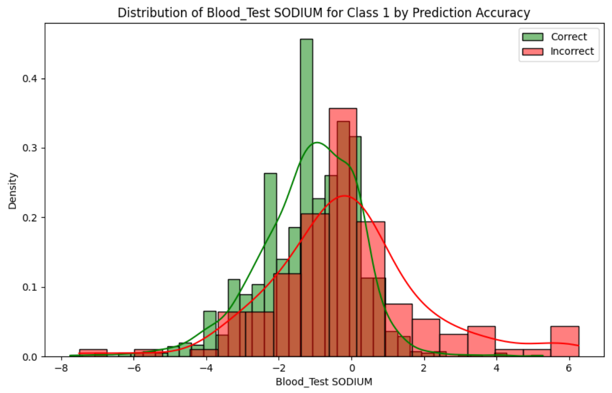
Distribution of Blood_Test SODIUM for Class 1 by Prediction Accuracy
Correct
Incorrect
Density
Blood_Test SODIUM

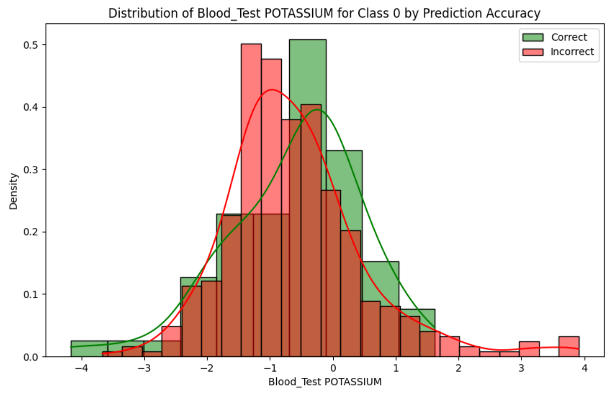
Distribution of Blood_Test POTASSIUM for Class 0 by Prediction Accuracy
Correct
Incorrect
Density
Blood_Test POTASSIUM

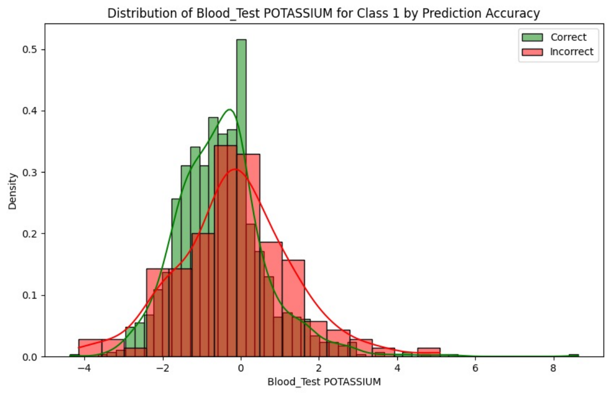
Distribution of Blood_Test POTASSIUM for Class 1 by Prediction Accuracy
Correct
Incorrect
Density
Blood_Test POTASSIUM

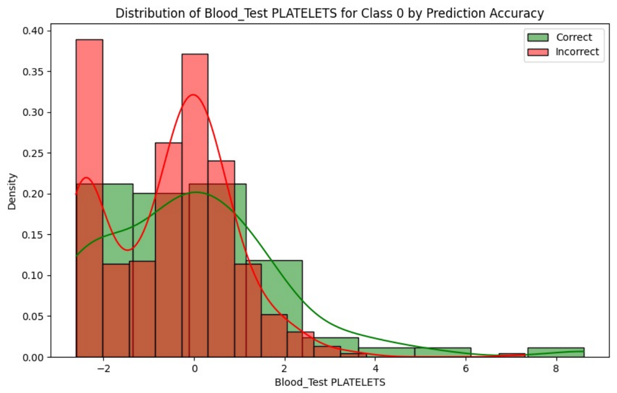
Distribution of Blood_Test PLATELETS for Class 0 by Prediction Accuracy
Correct
Incorrect
Density
Blood_Test PLATELETS

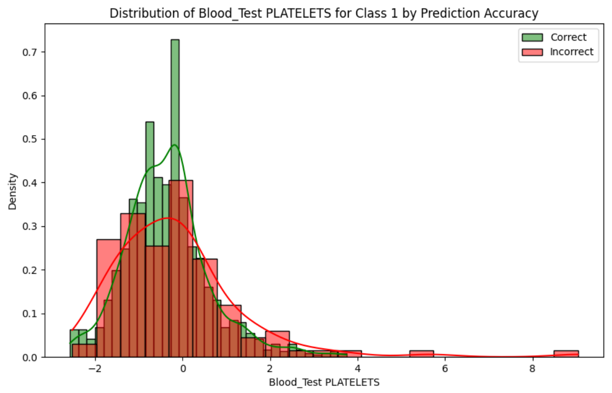
Distribution of Blood_Test PLATELETS for Class 1 by Prediction Accuracy
Correct
Incorrect
Density
Blood_Test PLATELETS

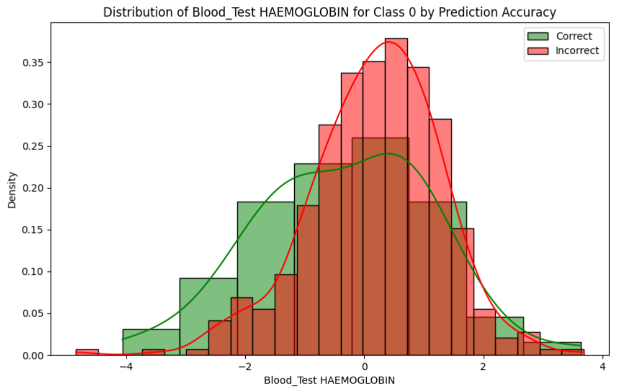
Distribution of Blood_Test HAEMOGLOBIN for Class 0 by Prediction Accuracy
Correct
Incorrect
Density
Blood_Test HAEMOGLOBIN

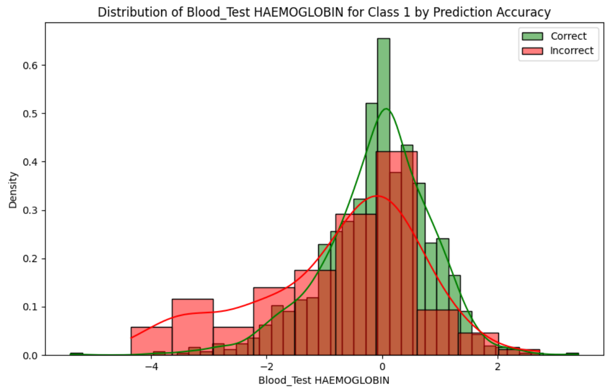
Distribution of Blood_Test HAEMOGLOBIN for Class 1 by Prediction Accuracy
Correct
Incorrect
Density
Blood_Test HAEMOGLOBIN

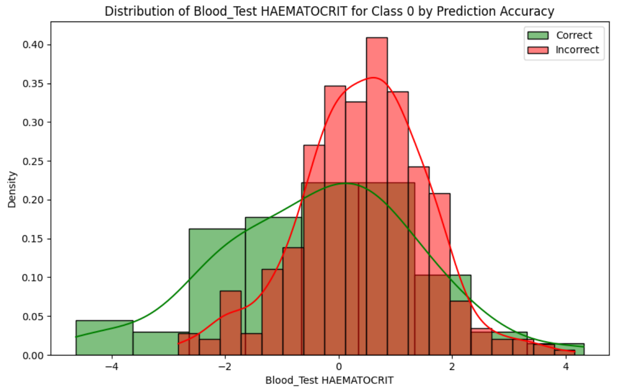
Distribution of Blood_Test HAEMATOCRIT for Class 0 by Prediction Accuracy
Correct
Incorrect
Density
Blood_Test HAEMATOCRIT

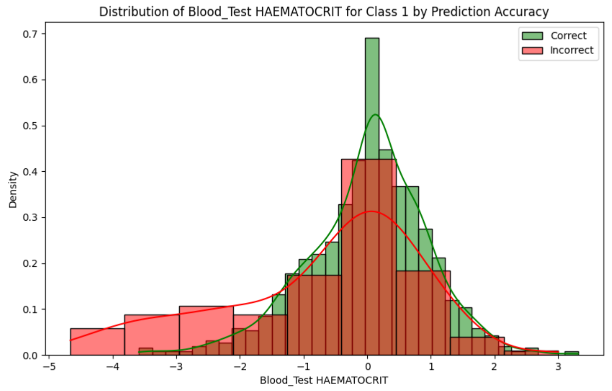
Distribution of Blood_Test HAEMATOCRIT for Class 1 by Prediction Accuracy
Correct
Incorrect
Density
Blood_Test HAEMATOCRIT

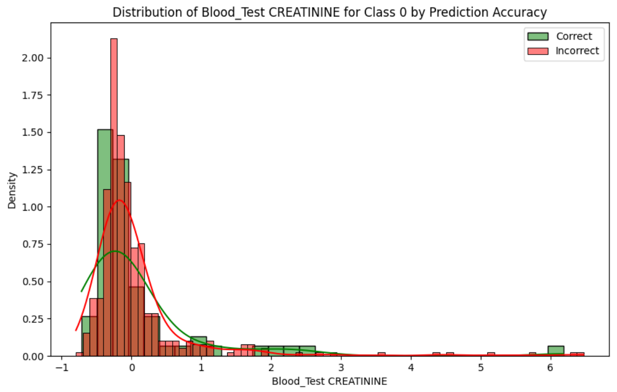
Distribution of Blood_Test CREATININE for Class 0 by Prediction Accuracy
Correct
Incorrect
Density
Blood_Test CREATININE

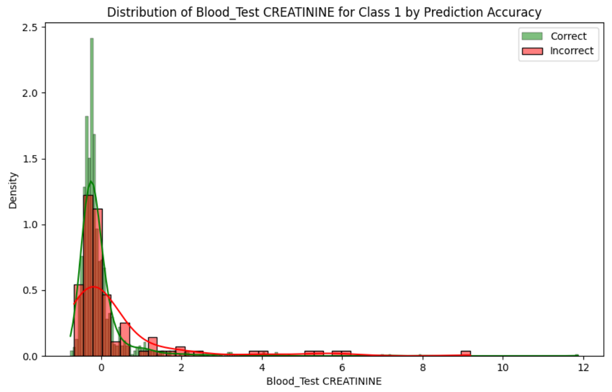
Distribution of Blood_Test CREATININE for Class 1 by Prediction Accuracy
Correct
Incorrect
Density
Blood_Test CREATININE

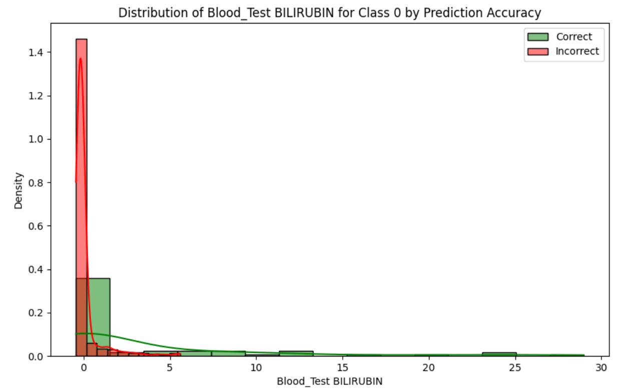
Distribution of Blood_Test BILIRUBIN for Class 0 by Prediction Accuracy
Correct
Incorrect
Density
Blood_Test BILIRUBIN

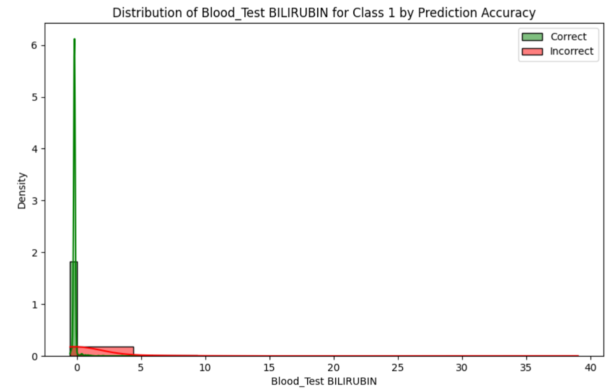
Distribution of Blood_Test BILIRUBIN for Class 1 by Prediction Accuracy
Correct
Incorrect
Density
Blood_Test BILIRUBIN

NHTD – outcome distributions
Neural Network, reduced feature set

Class 0 – COVID-negative

Class 1 – COVID-positive

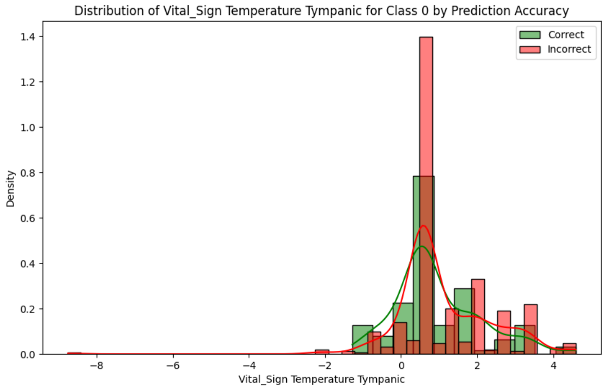

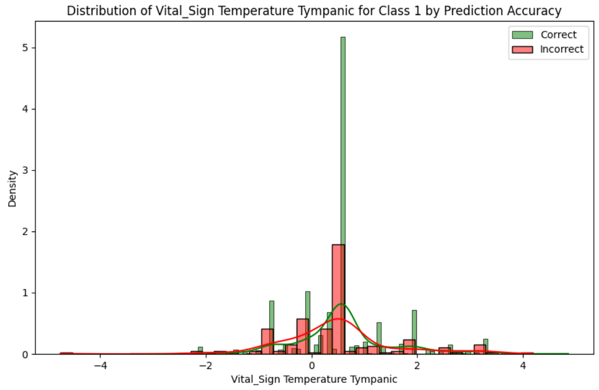

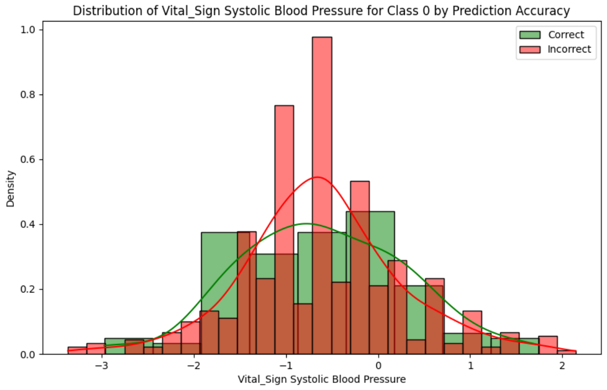

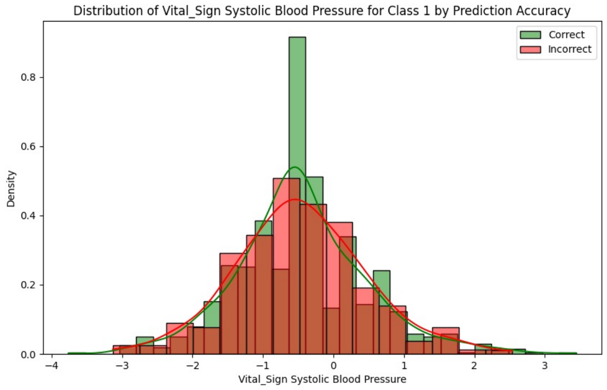

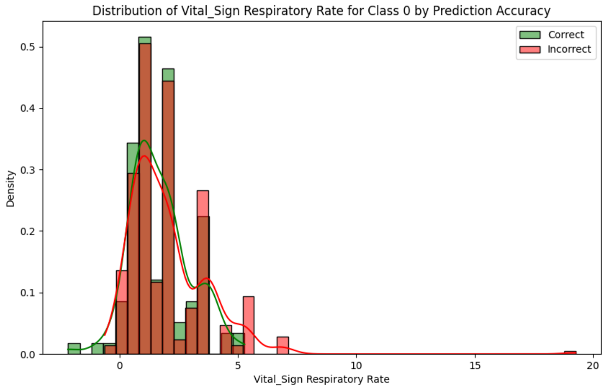

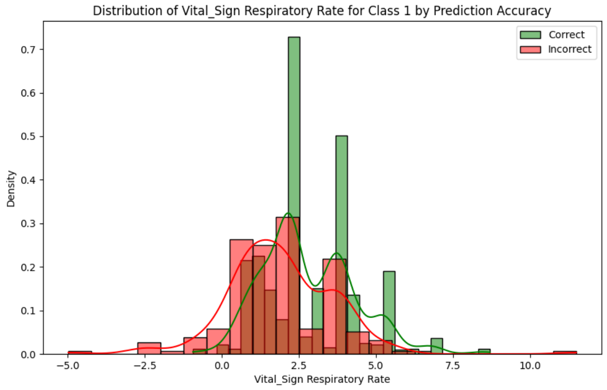

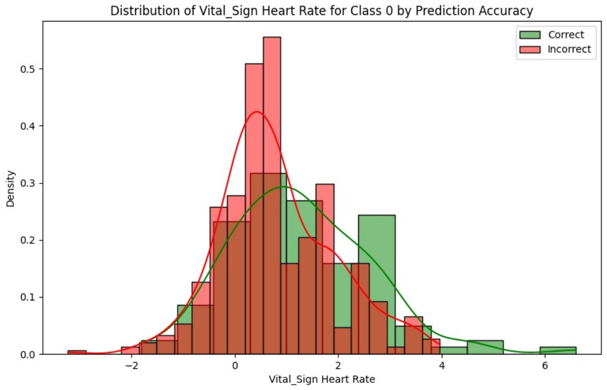

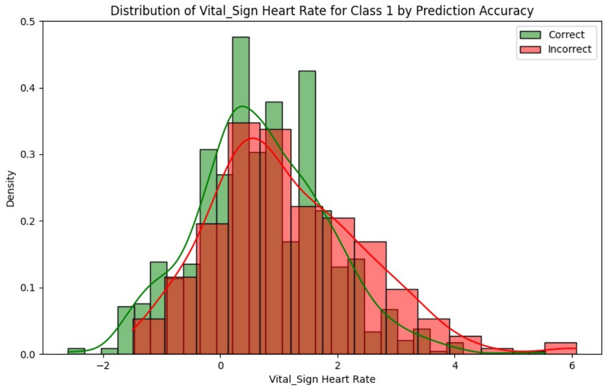

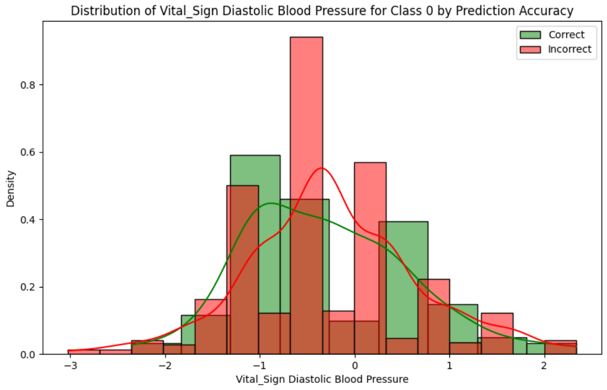

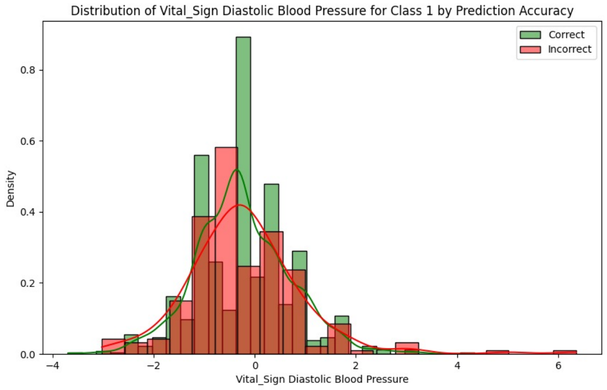

E References

[1] Soltan, A. A., Yang, J., Pattanshetty, R., Novak, A., Yang, Y., Rohanian, O., ... & Muthusami, V. (2022). Real-world evaluation of rapid and laboratory-free COVID-19 triage for emergency care: external validation and pilot deployment of artificial intelligence driven screening. The Lancet Digital Health, 4(4), e266-e278.

[2] Yang, J., Soltan, A. A., & Clifton, D. A. Machine learning generalizability across healthcare settings: insights from multi-site COVID-19 screening. npj Digital Medicine, 5(1), 69. (2022).

[3] Yang, J., Soltan, A. A., Eyre, D. W., Yang, Y., & Clifton, D. A. An adversarial training framework for mitigating algorithmic biases in clinical machine learning. npj Digital Medicine, 6(1), 55. (2023).
