# Supplementary material for: Generalizability assessment of AI models across hospitals in a low-middle and high income country
Source: Nat Commun. 2024 Sep 27;15:8270. doi: 10.1038/s41467-024-52618-6 (PMC11436917; doi:10.1038/s41467-024-52618-6)
Supplement: Supplementary file 2 — Reporting Summary [file 41467_2024_52618_MOESM2_ESM.pdf]

nature portfolio

Corresponding author(s): Jenny Yang

Last updated by author(s): July 20, 2024



# Reporting Summary

Nature Portfolio wishes to improve the reproducibility of the work that we publish. This form provides structure for consistency and transparency in reporting. For further information on Nature Portfolio policies, see our Editorial Policies and the Editorial Policy Checklist.

## Statistics

For all statistical analyses, confirm that the following items are present in the figure legend, table legend, main text, or Methods section.

| n/a | Confirmed | |
|---|---|---|
| ☐ | ☒ | The exact sample size (*n*) for each experimental group/condition, given as a discrete number and unit of measurement |
| ☐ | ☒ | A statement on whether measurements were taken from distinct samples or whether the same sample was measured repeatedly |
| ☐ | ☒ | The statistical test(s) used AND whether they are one- or two-sided<br>*Only common tests should be described solely by name; describe more complex techniques in the Methods section.* |
| ☒ | ☐ | A description of all covariates tested |
| ☐ | ☒ | A description of any assumptions or corrections, such as tests of normality and adjustment for multiple comparisons |
| ☐ | ☒ | A full description of the statistical parameters including central tendency (e.g. means) or other basic estimates (e.g. regression coefficient) AND variation (e.g. standard deviation) or associated estimates of uncertainty (e.g. confidence intervals) |
| ☐ | ☒ | For null hypothesis testing, the test statistic (e.g. $F$, $t$, $r$) with confidence intervals, effect sizes, degrees of freedom and $P$ value noted<br>*Give P values as exact values whenever suitable.* |
| ☒ | ☐ | For Bayesian analysis, information on the choice of priors and Markov chain Monte Carlo settings |
| ☐ | ☒ | For hierarchical and complex designs, identification of the appropriate level for tests and full reporting of outcomes |
| ☒ | ☐ | Estimates of effect sizes (e.g. Cohen's $d$, Pearson's $r$), indicating how they were calculated |

*Our web collection on statistics for biologists contains articles on many of the points above.*

## Software and code

Policy information about availability of computer code

| | |
|---|---|
| Data collection | No software was used to collect data. |
| Data analysis | Experiments were performed using Python (v3.8.3). All models were run using an Intel Xeon E-2146G Processor (CPU: 6 cores, 4.50 GHz max frequency). Logistic regression was implemented using Scikit-learn (v1.3.2). The XGBoost classifier was implemented using the XGBoost package (v2.0.3). Neural network models were implemented using Pytorch (2.1.2+cu121). Code can be found in: https://github.com/yangjenny/standard_algorithms |

For manuscripts utilizing custom algorithms or software that are central to the research but not yet described in published literature, software must be made available to editors and reviewers. We strongly encourage code deposition in a community repository (e.g. GitHub). See the Nature Portfolio guidelines for submitting code & software for further information.

## Data

Policy information about availability of data

All manuscripts must include a data availability statement. This statement should provide the following information, where applicable:

- Accession codes, unique identifiers, or web links for publicly available datasets
- A description of any restrictions on data availability
- For clinical datasets or third party data, please ensure that the statement adheres to our policy

Data from OUH studied here are available from the Infections in Oxfordshire Research Database (https://oxfordbrc.nihr.ac.uk/research-themes/modernising-medical-microbiology-and-big-infection-diagnostics/infections-in-oxfordshire-research-database-iord/), subject to an application meeting the ethical and

governance requirements of the Database. Data from UHB, PUH and BH are available by direct request to the hospitals, subject to HRA and research \& governance approvals at the individual Trusts. These raw datasets are protected and are not publicly available due to data privacy regulations.

Data from HTD and NHTD are available through a managed access policy at OUCRU, through the CCAA Vietnam Data Access Committee, subject to an application meeting the ethical and governance requirements. The data sharing policy can be found here: https://www.oucru.org/data-sharing-policy/. These raw datasets are protected and are not publicly available due to data privacy regulations. Please contact Dr. Louise Thwaites (lthwaites@oucru.org) if you would like help accessing the data.

Source data for result Tables and Figures are provided with this paper.

## Research involving human participants, their data, or biological material

Policy information about studies with human participants or human data. See also policy information about sex, gender (identity/presentation), and sexual orientation and race, ethnicity and racism.

| | |
|---|---|
| Reporting on sex and gender | This was not relevant in the study, as we used clinical features such as blood tests and vital signs, and tested all patients (regardless of demographic groupings). |
| Reporting on race, ethnicity, or other socially relevant groupings | This was not relevant in the study, as we used clinical features such as blood tests and vital signs, and tested all patients (regardless of demographic groupings). |
| Population characteristics | The OUH pre-pandemic cohort had a median (IQR) age of 60 (38–76), OUH wave 2 was 72 (55–82), PUH was 69 (48–82), UHB was 63 (42–79), BH was 68 (48–82), HTD was 58 (43-71), and NHTD was 64 (47-76). The OUH pre-pandemic cohort had a female to male ratio of 53:47, OUH wave 2 had 46:54, PUH 45:55, UHB 53:47, BH 47:53, HTD 49:51, and NHTD 40:60. |
| Recruitment | We used retrospective data available, and thus, did not need to recruit participants. |
| Ethics oversight | In this study, we used clinical data with linked, deidentified demographic information for patients across hospital centres in the UK and Vietnam. United Kingdom National Health Service (NHS) approval via the national oversight/regulatory body, the Health Research Authority (HRA), has been granted for use of routinely collected clinical data to develop and validate artificial intelligence models to detect Covid-19 (CURIAL; NHS HRA IRAS ID: 281832). The study was limited to working with deidentified data, and extracted retrospectively; thus, explicit patient consent for use of the data was deemed to not be required, and is covered within the HRA approval. All necessary consent has been obtained and the appropriate institutional forms have been archived.<br><br>The ethics committees of the Hospital for Tropical Diseases (HTD) and the National Hospital for Tropical Diseases (NHTD) approved use of the HTD and NHTD datasets for COVID-19 diagnosis, respectively. The study was limited to working with deidentified data and collected as part of ongoing audit; thus, OxTREC (Oxford Tropical Research Ethics Committee), NHTD and HTD ethics committees have waived the need for individual informed consent for this process. All methods were carried out in accordance with relevant guidelines and regulations. |

Note that full information on the approval of the study protocol must also be provided in the manuscript.

# Field-specific reporting

Please select the one below that is the best fit for your research. If you are not sure, read the appropriate sections before making your selection.

- [x] Life sciences
- [ ] Behavioural & social sciences
- [ ] Ecological, evolutionary & environmental sciences

For a reference copy of the document with all sections, see nature.com/documents/nr-reporting-summary-flat.pdf

# Life sciences study design

All studies must disclose on these points even when the disclosure is negative.

| | |
|---|---|
| Sample size | We used a total of 191,312 patients in training, continuous validation, and testing of machine learning models. |
| Data exclusions | For the NHS data, we excluded patients without confirmatory PCR testing. For HTD and NHTD datasets, we included all patients. |
| Replication | We validated on 6 independent cohorts. We bootstrapped our method (using 1000 iterations) to obtain appropriate confidence intervals for all outcomes. |
| Randomization | Allocation of patients for training and validation were randomized and stratified by COVID-19 diagnosis. |
| Blinding | Blinding is not applicable, as this study compared machine learning results across all patients. |

# Reporting for specific materials, systems and methods

We require information from authors about some types of materials, experimental systems and methods used in many studies. Here, indicate whether each material, system or method listed is relevant to your study. If you are not sure if a list item applies to your research, read the appropriate section before selecting a response.

## Materials & experimental systems

| n/a | Involved in the study |
|---|---|
| ☒ | ☐ Antibodies |
| ☒ | ☐ Eukaryotic cell lines |
| ☒ | ☐ Palaeontology and archaeology |
| ☒ | ☐ Animals and other organisms |
| ☐ | ☒ Clinical data |
| ☒ | ☐ Dual use research of concern |
| ☒ | ☐ Plants |

## Methods

| n/a | Involved in the study |
|---|---|
| ☒ | ☐ ChIP-seq |
| ☒ | ☐ Flow cytometry |
| ☒ | ☐ MRI-based neuroimaging |

# Clinical data

Policy information about clinical studies

All manuscripts should comply with the ICMJEguidelines for publication of clinical research and a completedCONSORT checklist must be included with all submissions.

| | |
|---|---|
| Clinical trial registration | NA |
| Study protocol | NA |
| Data collection | This is a retrospective study, thus data was requested from respective hospitals after ethics approval. |
| Outcomes | We evaluate machine learning models for diagnosis of COVID-19 across 5 independent hospitals. |
